# Supplementary material for: Learning curve and surgical outcome of robotic assisted colorectal surgery with ERAS program
Source: Sci Rep. 2022 Nov 29;12:20566. doi: 10.1038/s41598-022-24665-w (PMC9709162; doi:10.1038/s41598-022-24665-w)
Supplement: Supplementary file 2 — Supplementary Information 2. [file 41598_2022_24665_MOESM2_ESM.zip › RAL Raw data-Ñ[▒K/139/OP_ñw╝╨░O▒Kñσ.pdf]

正式摘要(電子簽章：000000233672853)

W83 / W83

臺中榮民總醫院

Operation Note

[redacted]

- 手術日期：2022/01/05
- 手術醫師：VS 林俊余，R 陳威遠，R 陳凱祥，R 陳怡豪
- Anesthesia : GE
- Position : Lithotomy
- Aseptic agents : Soap , Betadine Alcohol , Betadine Aqua

Diagnosis

- Pre-operative diagnosis : rectal cancer s/p SCRT
- Post-operative diagnosis : Ditto.

Surgical Indication

- Tumor resection

Operation

- Robot Assisted Low Anterior Resection

Finding

- 1. ulcerative tumor, about 3x3 cm in circumferential 5 cm FAV posterior wall.
  2. Total meso excision was complet
  3. IMA high ligation+ ICG+ . Proximal perfusion: 30sec. Distal perfusion: 25 sec.
  4. distal margin 1 cm, Stapler 45mm x4 for rectum
  5. air tie test neg. donut intact. H/V in pelvis.
  6. loop ileostomy in RLQ
  7. anastomosis re-enforce with V-lock continous suture.

Procedure

- 1. under GE, the pt was in lithotomy position
  2. the trocar hold was made in periumbilicus open method. the other 5 hole made by punctiure
  3. identify holly plain and dissct. isolated IMA root. ligated with clips
  4. dissect between Gerota fascia and meso rectum. lysis Lt white line
  5. dissect into pelvis area. dissect posterior side of rectum down to pelvis follor levetar ani. anterior side of Denovillor fascia. Total meso excision was complet
  6. resected rectum stump with Stapler 45mm 4 fires.
  7. pull the specimen outside abdomen from RLQ wd
  8. resect Rectum after check ICG level
  9. embeded envril 29 mm
  10.reset daVinci and complete anastomsis. air tie test neg. donut intact. H/V in pelvis.
  11. loop ileostomy in RLQ
  12.she tolerate whole procedure and extubated.
- Estimated blood loss: 100 c.c
- 器械數量: 正確
- 紗布數量: 正確

Image

- PAINT
  - abdomen

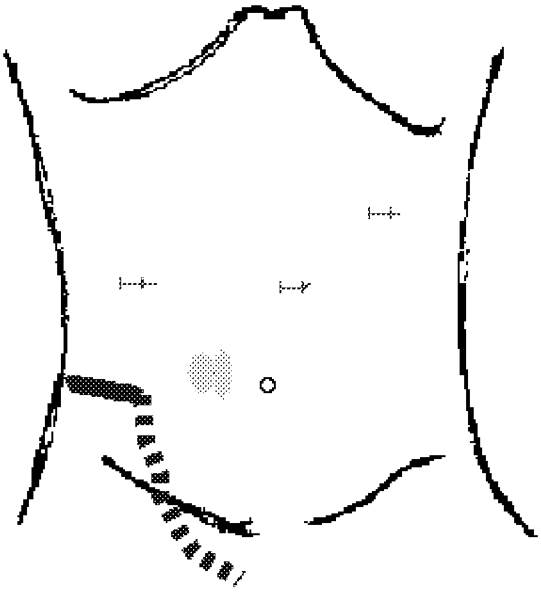

- colon & rectum

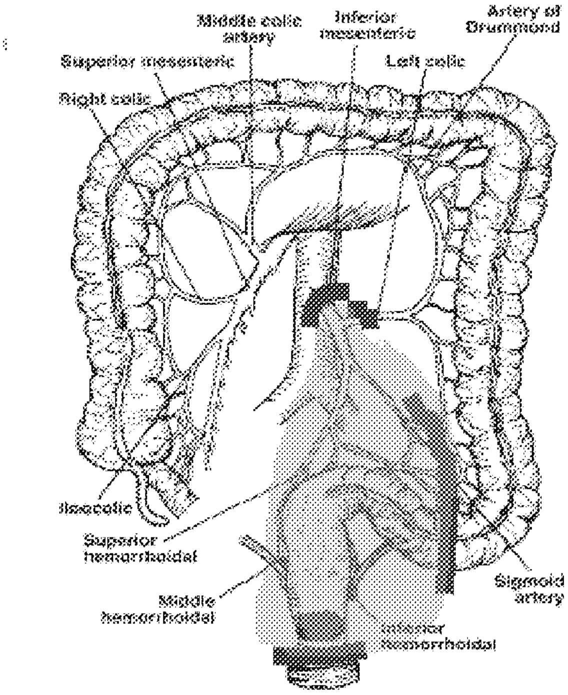

主治 2222H林俊余 / 醫師 2292G楊曙榮

正式摘要**(電子簽章：000000233672853)**

W83 / W83

doctorRank:B
